# Complete-Coverage Path-Planning Algorithm Based on Transition Probability and Learning Perturbation Operator

**DOI:** 10.3390/s25113283

**Published:** 2025-05-23

**Authors:** Xia Wang, Gongshuo Han, Jianing Tang, Zhongbin Dai

**Affiliations:** 1 School of Electrical and Information Technology, Yunnan Minzu University, Kunming 650504, China; wangxiacsu@163.com (X.W.); 18032599471@163.com (G.H.); 2 Yunnan Key Laboratory of Unmanned Autonomous System, Yunnan Minzu University, Kunming 650504, China; 3 Nanjing Branch of China Telecom Co., Ltd., Nanjing 210000, China; daizb@chinatelecom.cn

**Keywords:** complete-coverage path-planning, transition probability, initialization strategy, population hierarchy, perturbation and learning

## Abstract

To achieve shorter path length and lower repetition rate for robotic complete coverage path planning, a complete-coverage path-planning algorithm based on transition probability and learning perturbation operator (CCPP-TPLP) is proposed. Firstly, according to the adjacency information between nodes, the distance matrix and transition probability matrix of the accessible grid are established, and the optimal initialization path is generated by applying greedy strategy on the transition probability matrix. Secondly, the population is divided into four subgroups, and different degrees of learning perturbation operations are carried out on subgroups to update each path in the population. CCPP-TPLP was tested against five algorithms in different map environments and in the working map environment of electric tractors with height information The results show that CCPP-TPLP can optimize the selection of path nodes, reduce the total length and repetition rate of the path, and significantly improve the planning efficiency and quality of complete coverage path planning.

## 1. Introduction

In recent years, with the rapid development of intelligent technology and the continuous promotion of industrial wisdom upgrading, intelligent mobile robots have been widely used in more and more fields, such as cargo handling, intelligent production, intelligent life, abnormal environment detection, underwater operation, space exploration and so on [1]. Complete coverage path planning (CCPP) is a key research area in modern robotics, aiming to enable the mobile robot find a collision-free shortest path in a specific environment, meanwhile, it should traverse the entire accessible working area to form a continuous path that encompasses all accessible areas. In CCPP research, based on environmental characteristics, it can be categorized into CCPP problems in static environments and that in dynamic scenarios. Although dynamic scenarios are more common in real-world environments, there are also numerous practical applications in static scenarios. Examples include industrial robotic arms planning coverage paths on fixed workpieces for uniform welding or spraying tasks, automated guided vehicles (AGVs) optimizing paths in warehouses with fixed shelf layouts to cover all cargo access areas, and robots following fixed orchard tree distribution paths for pruning, harvesting, or monitoring. In these scenarios, the environment is typically fixed. Research on static CCPP methods can optimize efficiency and reduce time and energy consumption. Additionally, studies on static environments serve as a foundation for dynamic environment research. Many algorithms for dynamic environments require first addressing basic path planning in static environments before incorporating obstacle avoidance and real-time adjustment modules. If algorithms for static environments are inefficient, their performance in dynamic environments will also be affected.

CCPP algorithms can be categorized into three categories, classical algorithms, heuristic-based algorithms and hybrid algorithm. Classical algorithms are mainly as follows: the random walk algorithm [2], chaotic coverage path planner [3], the boustrophedon coverage algorithm [4], the interior screw algorithm [5], and the domain decomposition algorithm [6]. These algorithms are easy to operate and the calculation speed is fast; however, when it comes to practical application, these algorithms have poor efficiency and a high coverage repetition ratio for the planned path [7]. To solve the above problems, many scholars have improved the classical algorithms. Hou et al. [8] divide the target coverage area into boundary and center regions for separate coverage. The boundary region is covered using a spiral path, which do not generate overlapping; in the center region, two steering paths with low overlap rates are designed, to solve the problem of existing spiral CCPP algorithms not meeting the kinematic constraints of robots and have a high overlap rate in the center region. Chaotic path planning is a prospective modern approach that use chaotic dynamical systems to generate trajectories throughout an environment. Moysis et al. [9] proposed a chaotic path planning method for 3D area coverage using modified Logistic map and a modulo tactic. An area-coverage path planning which is controlled by a novel memristor-based double-stroll chaotic system has been proposed in [10] to generate higher unpredictability, more even and faster scanning path. Chaotic CPP ensures high coverage efficiency in the entire workspace in terms of the robot’s trajectory, guaranteeing faster coverage in the working space because the motion is pre-determined [11].

With the rapid advancement of technologies associated with artificial intelligence, heuristic algorithms have been increasingly and extensively applied in CCPP. By integrating intelligent optimization strategies, this type of algorithms can effectively address the challenge of CCPP in complex environments. It particularly demonstrates substantial advantages in scenarios characterized by dynamic environments, multiple constraints, and high real-time requirements. It mainly includes A* algorithm [12], D* algorithm [13], Theta* algorithm [14], evolutionary algorithms, swarm intelligence algorithms, the biologically inspired neural network algorithms and reinforcement learning, etc. In [15], the objective function is optimized based on evolutionary algorithms such as the genetic algorithm (GA) and ant colony optimization (ACO) of the traveling salesman problem (TSP) and estimates the shortest path that connects all waypoints. Considering full-load travel distance, unloading position distribution and the area of the turning area simultaneously, a full coverage path planning methods of harvesting robot with multi-objective constraints is proposed by using the shuttle method and the improved ACO algorithm [16]. The biologically inspired neural network algorithms do not require training the model, nor do they need to predict environmental information, and have a relatively fast computing speed. Therefore, it has been widely studied and applied by many scholars. In this algorithm, the map is shown as a grid map, and each grid is considered as a neuron. The adjacent neurons are connected, and the robot’s mobile path is chosen by calculating the neuronal activity value. Muthugala et al. [17] put forward an online path planning approach based on the Glasius Bio-inspired Neural network to enhance the energy efficiency and coverage effect of hull maintenance robots. By introducing a comprehensive energy model and a dynamic tracking method, this approach not only boosts the adaptability and energy efficiency in a dynamic environment but also strengthens the global coverage capability. Zhang et al. [18] brought in the domain neuron status criterion, which increased the biologically inspired path planning efficiency near isolated island obstacles. Li et al. [19] raised a 2D adaptive cell decomposition method, which strengthens the positive correlation between the obstacle density and the closure of the map grid. They incorporated the consideration of obstacle distribution and grid level into the bypass equation of the biologically informed neural networks (BINN) algorithm, thereby enhancing the efficiency and effectiveness of the CCPP for the bulldozer. On the other hand, the rapid development of reinforcement learning and deep learning brings a whole new solution to path planning. Elfoly et al. [20] proposed to use a modified Q-learning geometric learning algorithm to generate an optimal path by iteratively exploring and exploiting the state-action space. An improved deep reinforcement learning algorithm, the re-DQN algorithm, is proposed by Wang et al. [21] to solve the traversal order of each region. The reinforcement learning method possesses a strong ability for decision making and can find the global optimal solution; however, it costs much more time during the learning process than a generic algorithm, so its application has not been widely used [7].

Recent trends include the use of hybrid algorithm methods, combination of classical algorithms and heuristic algorithms, or combination of both heuristic algorithms to optimize CCPP solutions. Although the hybrid algorithm incurs a high computation time, it delivers better coverage efficiency. A convolutional neural network (CNN) with long short term memory (LSTM) layer was trained using the actor-critic experience replay (ACER) reinforcement learning algorithm in [22], and a complete area coverage planning module for the modified hTrihex, a honeycomb-shaped tiling robot, based on the deep reinforcement learning technique is proposed. Xu et al. [23] proposed a complete coverage neural network (CCNN) algorithm, which simplified the calculation process of neural activity. It also combined a modified A* algorithm to improve the covering efficiency. Tan et al. [7] presented a biologically inspired neural network algorithm based on Q-learning, which proceeds with path planning based on the biologically inspired neural network algorithm, and when it comes to the position change of a reachable path point, processes path optimization by adopting Q-learning to avoid falling into a locally optimal solution. Cheng et al. [24] proposed an improved PSO combined gray wolf optimization (IPSO-GWO) algorithm with chaos and a new adaptive inertial weight. Qian et al. [25] combined the interior screw search and the biologically inspired neural network algorithm, which reduced the calculating time of the path repetition ratio. Kvitko et al. [26] presented a path-planning algorithm based on the chaotic behavior of the Courbage–Nekorkin neuron model with a coverage control parameter to reduce the number of iterations required to cover the chosen investigated area.

Having said all of above, the current CCPP methods exhibit diverse characteristics when responding to the demands of different scenarios. Classical algorithms are known for their fast computing speed, but they are difficult to meet the requirements of efficient coverage due to the high path repetition rate. The A* algorithm and the D* algorithm are relatively sensitive to heuristic functions, and the computational overhead of the D* algorithm is relatively large. Evolutionary algorithms and swarm intelligence algorithms maintain the excellent global exploration performance of the Monte Carlo method and have a strong local exploitation capability. However, balancing exploration and development is a challenge, and there is a hidden danger of falling into local optimum. The self-learning and parallelism characteristics of the neural network algorithm can improve the coverage efficiency of the CCPP algorithm, while it can realize automatic obstacle avoidance and escape from the deadlocks [27]. however, it is more computationally intensive [19]. Moreover, there is no optimal value for parameters of the neural network model, which can only be determined by repeated experiments [28]. The continuous motion of chaotic CCPP enables the robot to move in searching and finding the target effectively with a more uniform coverage density. However, the existing literature only highlighted the coverage rate, ignoring the cost of coverage time. The unpredictable trajectory is also hugely dependent on the kinematic motion of the robot subjected to kinematic constraint, and it needs to be studied [11]. In addition, the notable dependence of the space coverage rate on the starting point of the autonomous robot’s path is the key shortcoming of chaos-based path-planning methods [26]. The hybrid algorithm attempts to balance efficiency and quality, but the increase in complexity and scene dependence have become new bottlenecks. Therefore, this paper proposes a complete-coverage path-planning algorithm based on transition probability and learning perturbation operator (CCPP-TPLP) to achieve efficient path planning within a concise algorithm framework. In the initialization stage of the algorithm, a greedy initialization strategy based on transition probability is proposed to generate high-quality initial solutions. The creation process of each solution takes both the adjacent properties of the grid and the requirement of the shortest path distance into account, ensuring that each initial solution is approximate optimal and laying a solid foundation for the subsequent optimization of the algorithm. A learning perturbation operator is proposed for algorithm iteration, thereby enhancing the diversity of the population and the convergence speed. Simulation results show that compared with five representative optimization algorithms, CCPP-TPLP achieves the best effect of complete coverage path planning under various obstacle densities and map scales. In the CCPP problem of an electric tractor in complex agricultural terrain, the complete coverage optimal path with the lowest energy consumption, shortest path length, and lowest repetition rate can be acquired by CCPP-TPLP.

This paper is structured as follows: Section 1 presents the problem description of CCPP; Section 2 introduces the related distance calculation method; Section 3 describes the complete coverage path planning method based on transfer probability and learning perturbation operator; Section 4 validates the effectiveness of the proposed algorithm in this paper through the ablation experiments, algorithmic comparison experiments, and algorithmic simulation experiments in 3D maps of motorized tractors; and Section 5 concludes with the summary and discussion.

## 2. Description of the Problem

CCPP pertains to obtaining the shortest path that traverses all areas except for obstacles within a closed region. The commonly employed map construction methods include the grid method [29], the topological method [30], and the geometric method [31]. Among them, grid maps possess duality and can represent the occupancy of obstacles in the workspace, thus being widely utilized in robot path planning. In this paper, the grid method is employed to quantize the robot’s working environment into several grids. The obstacles are represented by regular and complete grids, and two state values of 0 and 1 are used to represent the open space grid and the obstacle grid respectively.

In the grid map, the size of the grid is set to the size of the robot’s own coverage area. It is assumed that once the robot passes through the center of the grid, the grid is regarded as having been covered. The grid map consists of rows and columns. The row and column coordinates corresponding to the i-th grid are denoted by $\left(x_{i},y_{i}\right)$, where i represents the sequence number of the grid. Then the transformation relationship between i and grid coordinates is presented in Equation (1).

(1) $$i=(y_{i}-1)N+x_{i}$$

where N represents the number of grids contained in a column of the map. Let J={1,2,⋯,Κ} denote the set of all grid indices, and K represents the maximum number of grid. $T_{s}$ denote the set of subsets formed by picking s elements from the set J with replacement, which s=1,2,⋯K. If the set $C=J\cup T_{s}$, then the number of elements in C is K+s, and the total number of complete permutations for all elements in C is (K+s). Let $\sigma_{w}$ be a complete permutation of all elements in C. It is known that w∈[1,2,⋯,(K+s)!]. $X_{\sigma_{w}}$ represents a path formed by connecting the grids in $\sigma_{w}$ sequentially. Then, the mathematical model of the CCPP problem can be expressed as shown in Equation (2).

(2) $$\min F(X_{\sigma_{w}})$$

where $F(X_{\sigma_{w}})$ denotes the path length of the path $X_{\sigma_{w}}$. Since J already contains all grid indices, and $T_{s}$ is the set of subsets formed by picking s elements from {1,2,⋯,Κ} with replacement, in other words, every element of $T_{s}$ has already appeared once in J, thus, the smaller s is, the fewer overlapping elements exist between $T_{s}$ and J are, the less the number of repeated grids in the path is, and the lower repetition rate of the nodes in the path is.

## 3. Basic Theory

At present, the frequently employed distance calculation methods in the CCPP problem comprise Euclidean algorithm [32], Manhattan distance algorithm [33] nearest neighbor algorithm [34], Dijkstra algorithm [35], Floyd algorithm [36], and so forth. Among them, Euclidean algorithm and nearest neighbor algorithm are employed to calculate the straight-line distance between two points, regardless of the existence of obstacles. However, they do not give thought to the existence of obstacles. Manhattan distance algorithm merely reflects on the actual total moving distance from one point to another in the horizontal and vertical directions, without considering diagonal movement and the presence of obstacles. Dijkstra algorithm addresses the single-source shortest path problem, where each execution calculates the shortest paths from one starting node to all other nodes in the graph. While Floyd algorithm solves the all-pairs shortest path problem, computing the shortest paths between all node pairs in a single run. In the CCPP problem for grid maps, assuming the total number of grids is K, it is necessary to obtain the shortest distances between all nodes. If Dijkstra algorithm is used, the time complexity for a single execution is O(KlogK). Calculate the shortest distances between all nodes requires running Dijkstra algorithm once for each node as the starting point, resulting in a total time complexity of $O(K^{3}\log K)$. In contrast, Floyd algorithm computes all-pairs shortest paths in a single run with a fixed time complexity of $O(K^{3})$. Since Dijkstra algorithm exhibits significantly reduced computational efficiency in grid maps with a large number of nodes and may lead to local optima in complex scenarios, this work adopts Floyd algorithm to calculate the shortest distances between grids.

Floyd algorithm is a classic algorithm for addressing the shortest path problem in weighted networks, and its core lies in an idea of gradual approximation. By gradually incorporating intermediate nodes between any two nodes, the initial direct path distance between the two nodes is gradually extending to the path distance connected by multiple intermediate nodes, and the shortest path is chosen and retained throughout the process, so as to obtain the shortest distance between the two nodes. The basic steps are as follows.

(1) Initialize the distance matrix D

The distance matrix $D={\left[d_{i,j}\right]}_{K\times K}$ is constructed, where K is the number of nodes in the graph, and $d_{i,j}$ denotes the distance from grid i to grid j. If there is a direct edge connection between i and j, $d_{i,j}$ represents the weight of the edge. If there is no weight value, then $d_{i,j}=1$. If there is no direct edge connecting i and j, then $d_{i,j}=\infty$. Specifically, if i=j, then $d_{i,j}=0$.

(2) Update the distance matrix D

For each pair of grids i and j, and for each potential intermediate grid k, let k iterates from 1 to K to update the shortest distance between grids i and j. When the shortest distance between i and j can be attained via the intermediate node k, then $d_{i,j}$ is updated, and the update formula is Equation (3).

(3) $$d_{i,j}=\min\{d_{i,j},d_{i,k}+d_{k,j}\}\;i,j,k=1,2,\cdots ,K$$

After the traversal and iteration of all nodes, the distance matrix D is updated, and $d_{i,j}$ represents the shortest path from node i to node j.

## 4. CCPP Method Based on Transition Probability and Learning Perturbation Operator

### 4.1. Greedy Initialization Strategy Based on Transition Probability

In the path planning problem, if the population is randomly initialized, that is, a complete coverage path is randomly generated, the length of the initial path may be long, and too many nodes are visited repeatedly. Utilizing such a path as the initial one to participate in the subsequent iteration will significantly reduce the search efficiency of the algorithm. If the length of the initial path is short, the quality of the initial population can be improved. To this end, this paper proposes a greedy initialization strategy based on transition probability. At first, the map is transformed into a grid map. Then for all accessible grids, the shortest distance between any two grids is calculated by Floyd algorithm. Finally, during the path initialization process, the subsequent accessible grids are selected one by one from the starting grid. The grids closer to the current grid have a higher probability of being selected, thereby obtaining a short initial path.

The specific implementation procedures of the greedy initialization strategy based on transition probability are as follows.

(1) State matrix and adjacency matrix

**Figure 1:**
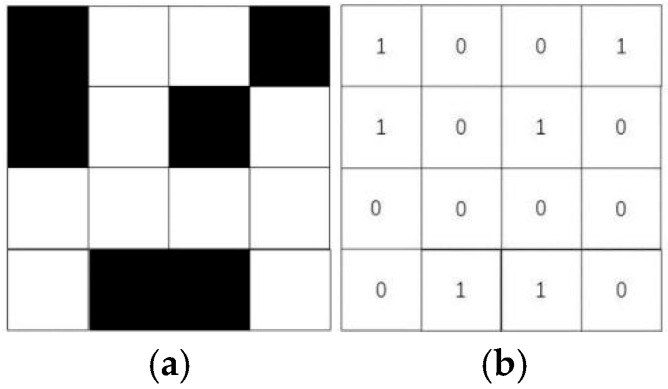
Grid map and its state matrix. (**a**) Schematic of the map rasterization. (**b**) State matrix.

The map is converted into a grid map, and it is assumed that the processed map contains *N* × *M* grids, as depicted in Figure 1a, where the black grids represent obstacles and the white grids represent accessible grids. The state matrix $M_{sta}$ of the map is constructed based on whether there is obstacle in the grid, as shown in Figure 1b.

$M_{sta}\left[n\right]\left[m\right]$ represents the element located in row n and column m of the state matrix $M_{sta}$, n=1,2,⋯,N, m=1,2,⋯,M. The state matrix $M_{sta}$ is constructed by Equation (4).

(4) $$M_{sta}[n][m]=\left\{\begin{array}{l} 0,\;\mathrm{the}\mathrm{accessible}\mathrm{grid}\\ 1,\;\mathrm{the}\mathrm{obstacle}\mathrm{grid} \end{array}\right.$$

Based on the state matrix $M_{sta}$, the adjacency matrix $M_{adj}$ of the accessible grids is further constructed. Suppose that there are K elements with a value of 0 in $M_{sta}$, that is, there are K accessible grids. The adjacency matrix $M_{adj}$ with size of K×K needs to be established, where the element located in row i and column j of $M_{adj}$ is denoted as $M_{adj}\left[i\right]\left[j\right](i=1,2\cdots K\;,j=1,2\cdots K)$ and represents the adjacent relationship between grid i and grid j. Suppose that the position indexes of grid i and grid j in the state matrix $M_{sta}$ are $\left(n_{i},m_{i}\right)$ and $\left(n_{j},m_{j}\right)$ respectively, the Manhattan distance between grid i and grid j is calculated by Equation (5) to determine their adjacent relationship, then the adjacency matrix $M_{adj}$ is constructed.

(5) $$M_{adj}[i][j]==\left\{\begin{array}{l} 1\;,\left|n_{i}-n_{j}\right|+\left|m_{i}-m_{j}\right|=1\\ 0\;,\left|n_{i}-n_{j}\right|+\left|m_{i}-m_{j}\right|=0\\ \infty ,\left|n_{i}-n_{j}\right|+\left|m_{i}-m_{j}\right|>1 \end{array}\right.$$

(2) Distance matrix and transition probability matrix

For all accessible grids, the adjacency matrix $M_{adj}$ and Floyd algorithm are employed to calculate the shortest distance between any two grids and obtain the distance matrix D, as shown in Equation (6).

(6) $$D=\left[\begin{array}{cccc} d_{1,1} & d_{1,2} & \cdots & d_{1,K}\\ d_{2,1} & d_{2,2} & \cdots & d_{2,K}\\ \cdots & \cdots & \cdots & \cdots \\ d_{K,1} & d_{K,2} & \cdots & d_{K,K} \end{array}\right]$$

Here, $d_{i,j}$ represents the shortest distance between grid i and grid j. Based on the distance matrix D, the transition probability matrix P is defined as presented in Equation (7).

(7) $$P=\left[\begin{array}{cccc} p_{1,1} & p_{1,2} & \cdots & p_{1,K}\\ p_{2,1} & p_{2,2} & \cdots & p_{2,K}\\ \cdots & \cdots & \cdots & \cdots \\ p_{K,1} & p_{K,2} & \cdots & p_{K,K} \end{array}\right]$$

where $p_{i,j}$ represents the probability of transfer from grid i to grid j, which is calculated in accordance with Equation (8).

(8) $$p_{i,j}=\frac{\frac{1}{d_{i,j}^{\lambda }}}{\sum_{s=1}^{K}\frac{1}{d_{i,s}^{\lambda }}}$$

where λ is defined as a greedy factor. The smaller $d_{i,j}$ is, the larger $p_{i,j}$ is, and the higher the probability that grid j will be selected when the next transfer grid is chosen from grid i.

(3) Population initialization of greedy strategy

Since there are K accessible grids, the individual of creating population is $X=[x_{1},x_{2},\cdots ,x_{K+s}]$, which represents a path covering all accessible grids, and its arbitrary dimension variable $x_{k}\in [1,K]$ is the sequence number of accessible grids. $x_{1}$ is the sequence number of specified starting grid. To complete the individual initialization, set the degree of greediness $\alpha_{g}$ and generate the random number $rand_{1}$. If $rand_{1}<\alpha_{g}$, grid $j^{*}$ is selected as $x_{2}$ in accordance with Equation (9).

(9) $$j^{*}=\underset{j}{\arg\max}\;p_{x_{1},j},\;j=1,2,\ldots ,K$$

Otherwise, the cumulative sum probability of each row in P is computed, and a accessible grid is chosen as $x_{2}$ through the roulette—wheel selection method.

According to the aforesaid method, the subsequent grid numbers are determined in turn to complete the initialization of the individual. Populations are created based on the population size and the dimensions of decision variables. For each individual initialization, random number $rand_{2}$ is generated. If $rand_{2}<\alpha_{g}$, an individual is generated based on the starting point and transition probability matrix P, using the above-mentioned greedy strategy. Otherwise, each decision variable is randomly generated to construct a random individual. By setting the degree of greediness $\alpha_{g}$, the algorithm can strike a balance between the greedy strategy and the random strategy. If $\alpha_{g}$ is low, the algorithm is more inclined towards the greedy strategy, which is conducive to finding high-quality solutions rapidly. While $\alpha_{g}$ is high, the algorithm prefers random selection, which is conducive to increasing the diversity of the population and avoiding premature convergence.

Through the greedy initialization strategy based on transition probability, a high-quality initial population can be constructed simply and effectively. The creation process of each individual takes into account the adjacent characteristics of grids and the requirement that the path length must be the shortest, ensuring that each individual is an approximately optimal solution.

### 4.2. The Operation of Learning and Perturbation

To update the population, this paper proposes a learning perturbation operator to enhance the diversity and convergence of the population and assist the algorithm in escaping the local optimum. The objective function of CCPP is the shortest path length, and the main factor influencing the path length is the selected sequence of accessible grids. When the selection order of the accessible grids varies, it will result in obvious differences in the path length. The individuals are classified into different subgroups based on their fitness. Subsequently, different degrees of learning or perturbation strategies are employed for individuals in different subgroups to alter the sequence of the accessible grid, thereby influencing the fitness value of each individual. The specific implementation procedures are as follows.

(1) Hierarchical division of the population

**Figure 2:**
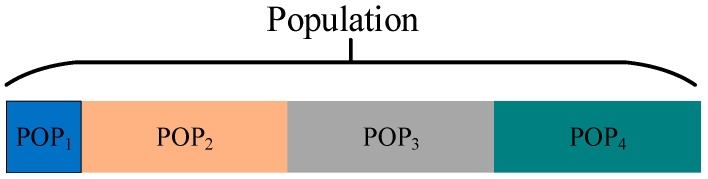
Schematic illustration of population hierarchy division.

The individuals within the population are managed in a hierarchical manner based on their fitness values. This hierarchy can assist the algorithm in exploring the solution space more effectively, enhance the search efficiency, and facilitate the maintenance of population diversity. In this paper, the individuals within the population are sorted based on the fitness value from small to large, and the individual possessing the best fitness value is classified into the first subgroup, namely, $POP_{1}=\left\{X_{best}\right\}$.The remaining individuals are categorized into three subgroups, namely $POP_{2}$, $POP_{3}$ and $POP_{4}$. The proportion of the hierarchy can be set according to the need. Different update strategies will be employed for individuals in different subgroups. Figure 2 presents a schematic illustration of the hierarchical division of the population, with different colors signifying different subgroups.

(2) Perturbation operation

Perturbation operation includes strong perturbation and weak perturbation. The aim of strong perturbation is to cause individuals to undergo significant random changes, thereby encouraging them to escape from the local optimal state as much as possible and explore a new solution space. While the weak perturbation is intended to decelerate the convergence process of the individual to the current optimum, thereby expecting to discover new and better solutions.

**Figure 3:**
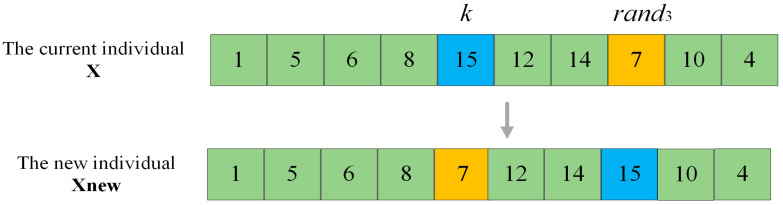
Strong perturbation operation. (The green grids in the figure are the unchanged ones, while the blue and orange grids are the changed ones.)

For $POP_{1}$, because of its optimal fitness value, it has a strong guiding effect on the entire population. To prevent the population from falling into the local optimum, the order of the accessible grid of the individual in $POP_{1}$ is randomly adjusted to cause a significant change in its fitness value, which is defined as the strong perturbation operation. Take Figure 3 as an example. Suppose that the current individual is x, and the accessible grid index to be operated is *k*. Firstly, random number *rand*_3_ within the range of [1,K] is generated. Then the accessible grid indexed by *rand*_3_ in x and the accessible indexed by *k* are swapped to complete the strong perturbation operation and obtain a new individual **X_new_**. As depicted in Figure 3, the calculation of the fitness value of x encompasses the shortest distance between grid 8 and grid 15, between grid 15 and grid 12, between grid 14 and grid 7, as well as between grid 7 and grid 10. However, for the perturbed individual **X_new_**, the above-mentioned four distances have transformed into the shortest distance between grid 8 and grid 7, between grid 7 and grid 12, between grid 14 and grid 15, and between grid 15 and grid 10. It can be observed that the strong perturbation operation alters the order of the accessible grids in the original individual to the greatest extent, and it will affect the shortest path among the four grids, thus having the greatest impact on the individual’s fitness value.

**Figure 4:**
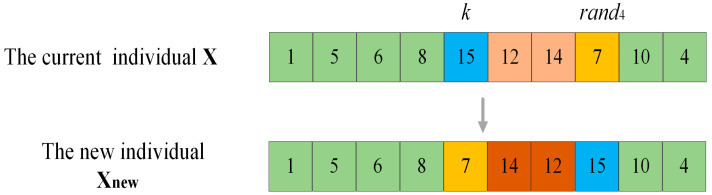
Weak perturbation operation. (The green grids in the figure are the unchanged ones, the blue and orange grids are the changed ones, and the grids of other colors are the ones that have undergone inverted changes.)

For $POP_{4}$, which is the farthest from the current optimum, in order to slow down its convergence towards the current optimum and thereby have the opportunity to discover new and more optimal solutions, it is operated with weak perturbation. Take Figure 4 as an example. Suppose that the current individual is X, and the accessible grid index to be operated is k. Generate a random number $rand_{4}$ between [1,K], all the accessible grids between the position of the index $rand_{4}$ in X and the position of the index k are reordered in reverse order to complete the weak perturbation operation, so as to obtain a new individual $X_{new}$. In Figure 4, the fitness value calculation of X encompasses the shortest distances between grid 8 and grid 15, as well as between grid 7 and grid 10. However, for $X_{new}$, the above two distances turn into the shortest distance between grid 8 and grid 7, and between grid 15 and grid 10. It can be observed that the change in the order of the accessible grid in the original individual resulting from the weak perturbation operation is less significant than that from the strong perturbation operation, for it only leads to the alteration of two distances. Therefore, the impact on the individual fitness value is minor.

(3) Learning operation

**Figure 5:**
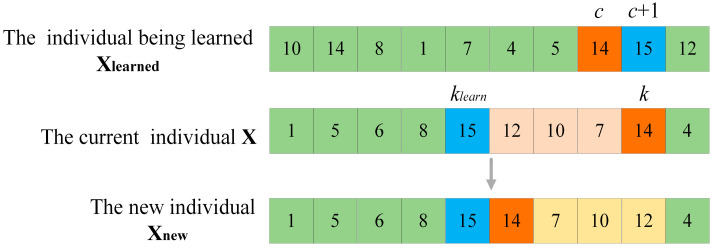
Learning operation. (The green grids in the figure are the unchanged ones, the blue and orange grids are the changed ones, and the grids of other colors are the ones that have undergone inverted changes.)

Learning operation includes learning from the optimal individual and learning from the random individual. For $POP_{2}$, to better maintain excellent genes, it should learn from the best individual $X_{best}$. However, for $POP_{3}$, to slow down its convergence to the current optimum, it is made to learn from random individuals within the population. Learning is accomplished through gene fragment learning, the specific learning operation is depicted in Figure 5. Suppose that the current individual is X, the individual being learned is $X_{learned}$, the index of the accessible grid to be operated in X is k, whose grid sequence number is $x_{k}$. In $X_{learned}$, locate index c, whose grid sequence number is also $x_{k}$, select the grid sequence number $x_{learned,c+1}$, whose index is c+1 in $X_{learned}$, and then return to the X to find the index $k_{learn}$, whose grid sequence number is $x_{learned,c+1}$ in X. Finally, all the accessible grids in X between the position of the index $k_{learn}$ and the position of the index k are reordered in reverse order, such that $x_{k}$ and $x_{k_{learn}}$ become adjacent accessible grids in $X_{new}$. It can be observed that through the learning operation, the individual can acquire part of the gene fragment from the learning object. If the learning object is $X_{best}$, the learned gene fragment might be an outstanding gene combination. And if the learning object is a random individual, the diversity of the individual can be enhanced.

### 4.3. Steps of the Algorithm

The implementation steps of the complete-coverage path-planning algorithm based on transition probability and learning perturbation operator are as follows.

Step 1: Define the iteration number t=1, the maximum iteration number $t_{max}$, the population size Num, the degree of greediness $\alpha_{g}$, and the greedy factor λ. Obtain the map information, set the starting and ending points of the path, and calculate the state matrix $M_{sta}$ of the map by means of Equation (4). The adjacency matrix $M_{adj}$ and distance matrix D of the map are respectively calculated by Equation (5) and Equation (6). The transition probability matrix D for each accessible grid transferring to other accessible grids is computed by Equation (7).

Step 2: Create an individual X, and utilize the specified starting grid number as the first element of X, i.e., $x_{1}$. Generate the random number $rand_{2}$. If $rand_{2}<\alpha_{g}$, then employ the greedy initialization strategy based on transition probability to generate the individual and proceed to Step 3. Otherwise, the individual X is randomly generated and proceed to Step 4.

Step 3: Generate the random number $rand_{1}$. If $rand_{1}<\alpha_{g}$, the next node in X is generated according to the transition probability matrix P and Equation (9). Otherwise, the cumulative sum probability of each row of P is calculated, and an accessible grid is selected as the next node in X by using the roulette—wheel selection method.

Step 4: Determine whether the individual X encompasses all accessible grids. If yes, go to Step 5. Otherwise, execute Step 3.

Step 5: If the population size reaches Num, Repair population to guarantee that the starting grids and end grids of all individuals meet the path requirements. Then Step 6 will be executed. If not, Step 2 will be executed and new individuals will be generated.

Step 6: If $t\geq t_{max}$, go to Step 9. Otherwise, go to Step 7.

Step 7: Arrange the fitness of all individuals in descending order and divide the population into four subgroups according to this order. Different learning or perturbation operations are carried out for individuals in different subgroups to generate a new population.

Step 8: Repair population to guarantee that the starting grids and end grids of all individuals meet the path requirements. Increase the number of iterations t by one and return to Step 6.

Step 9: Output the optimal solution $X_{best}$ of the population.

**Figure 6:**
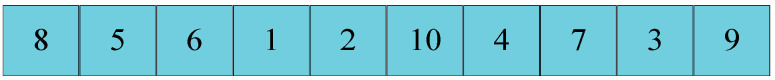
Disqualified path.

**Figure 7:**
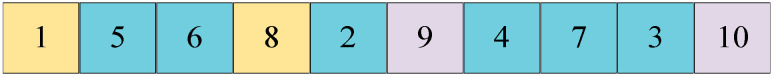
Qualified path. (The blue grids in the figure are the unchanged grids, the yellow part is the change caused by adjusting the starting point, and the purple part is the change caused by adjusting the endpoint.)

**Figure 8:**
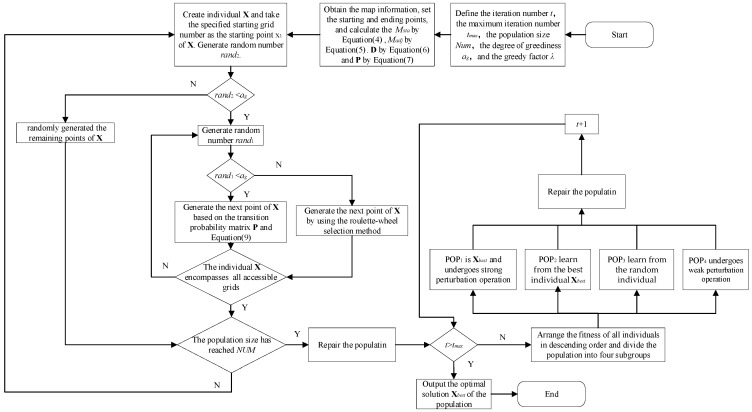
Flowchart of CCPP-TPLP algorithm.

The repair operation mentioned in Steps 5 and 8 is designed to ensure that the starting grids and ending grids of all individuals comply with the path requirement. For each individual in the population, it is necessary to guarantee that the path begins at the designated starting grid and terminates at the specified ending grid. As illustrated in the figure below, assuming the map’s starting point is grid 1 and the endpoint is grid 10, a generated path shown in Figure 6 does not start at grid 1 nor end at grid 10. This requires repair operations, which is swapping the position of grid 1 with the first node in the path and exchanging grid 10 with the last node in the path, as demonstrated in Figure 7. Through these repair operations, the starting grid is placed at the beginning, and the ending grid is positioned at the end of the path. Figure 8 presents the flowchart of the CCPP-TPLP algorithm.

## 5. Simulation Experiments and Analysis

In this section, the experimental setup is initially presented, including the experimental environment, the selected comparison algorithm, the settings of algorithm parameters, and the evaluation metrics. Then, the greedy initialization strategy based on the transition probability and the learning perturbation operator are verified independently. Finally, the algorithm is compared with representative optimization algorithms such as ant colony optimization (ACO) [37], grey wolf optimizer (GWO) [38], student psychology based optimization (SPBO) [39], discrete just another yet another (DJAYA) [40], and discrete tree seed algorithm (DTSA) [41] under different environment to verify their optimization effect on CCPP problem. In addition, to verify the algorithm’s capacity in solving practical issues, the CCPP problem of an electric tractor is addressed, and the solution outcomes of each algorithm are compared and analyzed.

### 5.1. Experimental Setting

(1) Experimental Environment

All experiments in this paper are performed on a PC with AMD Ryzen 7 5800H with Radeon Graphics, 3.20 GHz CPU, 16 GB RAM, and Windows 11 MATLAB R2021b.

(2) Comparing algorithm parameter settings

The parameter settings of the five algorithms selected in the comparative experiment are shown in Table 1.

(3) Evaluation indicators

The performance of the algorithm is evaluated by means of four indicators: coverage rate, coverage repeat rate, path length, and *Nic*.

Coverage rate

The coverage rate depicts the percentage of the area covered by the path. Since the entire map includes accessible area and obstacle area, the algorithm aims to achieve complete coverage of the accessible grid. The entire accessible area, the area covered by the path, and the obstacle area are respectively denoted as S, $S_{path}$, and $S_{obstacle}$, and the coverage rate (Cr) is defined by Equation (10).

(10) $$Cr=\frac{S_{path}}{S-S_{obstacle}}\times 100\%$$

Coverage repeat rate

The coverage repeat rate is the ratio of the area repeatedly covered in the path to the area of the accessible region. The definition formula of the regional repetition rate Crr is given in Equation (11).

(11) $$Crr=\frac{S_{repeat}}{S-S_{obstacle}}\times 100\%$$

Path length

The path length is also the objective function defined in Section 1.

The number of iterations to convergence (*Nic*)

*Nic* refers to the number of iterations when the algorithm converges.

(4) Map environment

**Figure 9:**
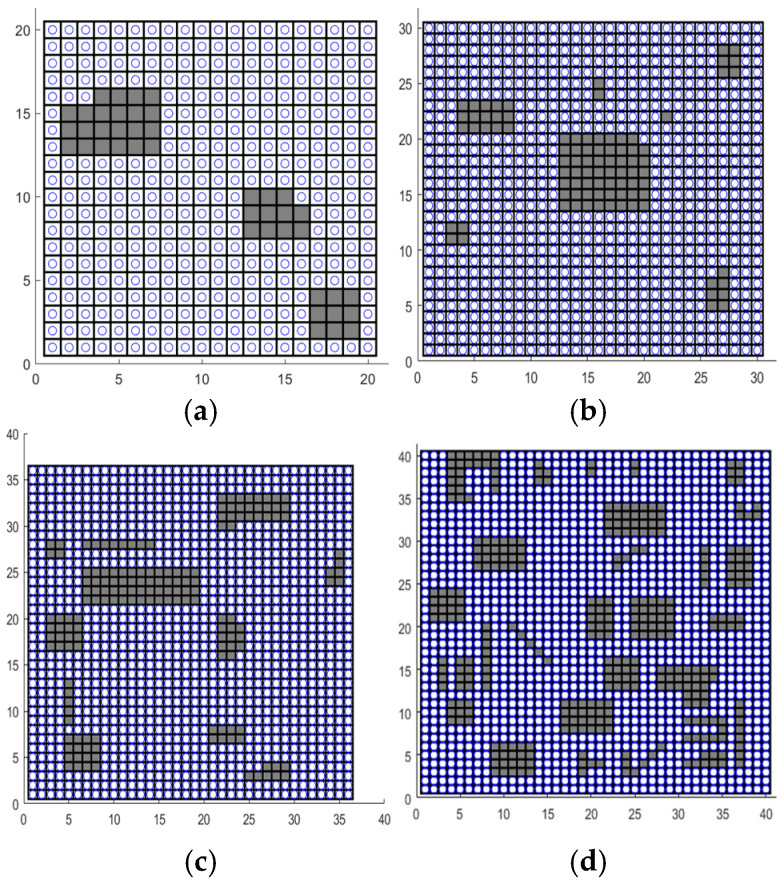
The schematic diagrams of four maps. (**a**) Environment I. (**b**) Environment II. (**c**) Environment III. (**d**) Environment IV. (The black parts represent obstacle grids, and grids containing circles represent accessible grids).

To comprehensively assess the performance of the CCPP-TPLP in diverse environments, four grid maps with different sizes and complexities are devised, whose complexity is characterized by three aspects: the map size, the proportion of accessible area, and the number of discrete obstacles. The proportion of accessible area represents the ratio of the number of accessible grids to the total number of grids in the entire map. The quantity of discrete obstacles represents the number of independent obstacles shown on the map. The specific parameters of the map environment are presented in Table 2. Parameters of four map environments, and the schematic diagrams of the maps are shown in Figure 9. 

### 5.2. Validation and Analysis of the Ablation Experiments

#### 5.2.1. Verification of the Greedy Initialization Strategy Based on Transition Probability

**Figure 10:**
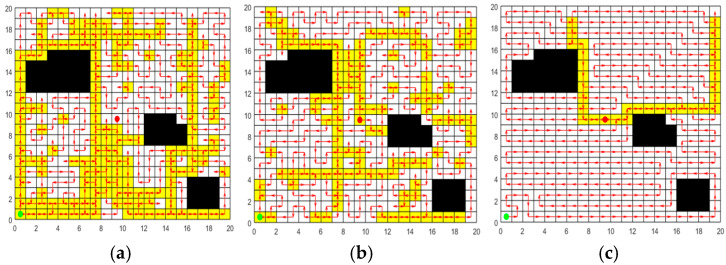
Initial paths generated by the three strategies. (**a**) Random initialization strategy. (**b**) Initialization strategy of ACO. (**c**) Greedy initialization strategy based on transition probability. (The green circle indicates the starting grid, the red circle represents the ending grid, the red arrow indicates the moving direction, the white grid represents the accessible grid, the yellow grid represents the repeated passing grids, and the black grid represents the obstacle grid. )

In order to verify the effectiveness of the greedy initialization strategy based on transition probability, the random initialization strategy and the initialization strategy of ACO are selected for comparison experiments. Set up a 20 m × 20 m two-dimensional map with 42 grids serving as obstacles for the experiment. The initialization paths are respectively generated by the random initialization strategy, the initialization strategy of ACO, and the greedy initialization strategy based on transition probability. The population size is set at 50, and each strategy is independently repeated 30 times for the experiment. In each experiment, the length of the shortest path in the initial population generated by each strategy is recorded. The 30 path results for each strategy are averaged to obtain the results in Table 3. The optimal paths generated by the three strategies are respectively plotted in Figure 10 where the green circle indicates the starting grid, the red circle represents the ending grid, the red arrow indicates the moving direction, the white grid represents the accessible grid, the yellow grid represents the repeated passing grids, and the black grid represents the obstacle grid. The more yellow grids there are, the higher the node repetition rate of the path is. From Figure 10 and Table 3, the following conclusions can be drawn:

(1) As can be seen from Figure 10 that the paths generated by the random initialization strategy and the initialization strategy of ACO contain numerous repeated passing grids, while the paths obtained by the greedy initialization strategy based on transition probability have relatively few repeated passing grids. This is because the random initialization strategy fails to take into account the relationship between grids, and the path constructed by it has strong randomness. Although ACO employs pheromones to guide the search process, in the initialization phase, the pheromone concentration on all paths is initialized to the same value, leading to a lack of guidance in the initial search. The greedy initialization strategy based on transition probability presented in this paper takes into account both the adjacent properties of grids and the requirement of the shortest path distance when selecting accessible grids, ensuring that the generated path length is shorter.
(2) It is observable from Table 3 that the initial paths of the three strategies can achieve 100% coverage. Among them, the greedy initialization strategy based on transition probability performs best in terms of path length and Crr, and can obtain initial paths with higher quality. Moreover, the standard deviation of the 30 paths obtained by this strategy is the smallest, indicating that this strategy demonstrates higher stability and reliability in multiple experiments.

In summary, through the comparative analysis of the three initialization strategies, the greedy initialization strategy based on transition probability demonstrates superior performance in terms of path length and Crr. This strategy effectively utilizes the distance and adjacency information between grids, significantly optimizes the selection of grids, reduces the repetition of paths, and thereby enhances the efficiency and quality of initial path planning.

#### 5.2.2. Validation of the Learning Perturbation Operation

**Figure 11:**
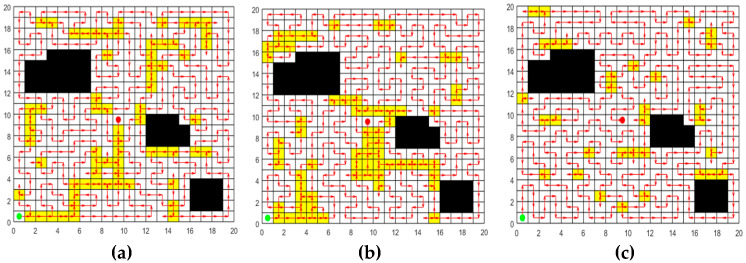
Optimal paths obtained by the three strategies. (**a**) The update strategy of ACO. (**b**) The update strategy of SPBO. (**c**) The update strategy of learning perturbation.

In order to validate the effectiveness of the learning perturbation operation, ACO and SPBO algorithms are chosen for comparative experiments. Set up a 20 m × 20 m two-dimensional map with 42 grids serving as obstacles for the experiment. The initial population is generated by the ACO algorithm, and the population size is 50. For the same initial population, ACO, SPBO, and learning perturbation operation strategy are respectively employed to update the population, and the number of update iterations is 100 as the same. Each strategy is independently repeated 20 times for the experiment. In each experiment, the length of the shortest path in the population updated by each strategy is recorded. The 20 path results for each strategy are averaged to obtain the results in Table 4. The optimal paths obtained by the three strategies are plotted in Figure 11 respectively.

Figure 11 and Table 4 display that the proposed learning perturbation operation has more advantages for population renewal. For the same initial population, through the update of the learning perturbation operation, the population with lowest Crr and path length can be obtained, and the standard deviation results of the above two indicators are the smallest, indicating that the stability of this strategy is also better.

#### 5.2.3. Impact of Parameters on Algorithm Performance

In order to verify the impact of the greedy factor λ, the degree of greediness $\alpha_{g}$ and different perturbation strategies on the performance of the CCPP-TPLP algorithm, set up a 20 m × 20 m two-dimensional map with 42 grids and a 30 m × 30 m two-dimensional map with 90 grids serving as obstacles for the experiment. Four perturbation strategies are defined as ① Both $POP_{1}$ and $POP_{4}$ adopt strong perturbation, ② Both $POP_{1}$ and $POP_{4}$ adopt weak perturbation, ③ $POP_{1}$ adopts weak perturbation and $POP_{4}$ adopts strong perturbation, and ④ $POP_{1}$ adopts strong perturbation and $POP_{4}$ adopts weak perturbation. The population size is 50, and each experiment of parameter value is independently repeated 10 times. The results of the optimal path found by the CCPP-TPLP algorithm under different parameter values or strategies are recorded and the 10 path results obtained under each parameter value or strategy are averaged to obtain the results in Table 5, Table 6 and Table 7.

It can be seen from Table 5, in Environment I, when λ is set to 5, the algorithm achieves optimal mean values of Crr, path length and *Nic* with the smallest standard deviations, indicating that the CCPP-TPLP algorithm attains the best performance in optimality, stability, and convergence. In Environment II, while the mean values of Crr, path length and *Nic* remain optimal when λ = 5, the standard deviations of Crr and path length are not minimized, suggesting that although the algorithm maintains the best performance in optimality and convergence, its stability is slightly compromised. Overall, the comprehensive performance of the CCPP-TPLP algorithm is optimal when λ is set to 5. Therefore, the value of the greedy factor λ is set to 5 in the subsequent experiments.

It can be known from Table 6, in Environment I, when $\alpha_{g}$ is set to 1, the algorithm’s Crr, path length and *Nic* achieve the optimal mean values with the smallest standard deviations, indicating that the CCPP-TPLP algorithm reaches the best performance in optimality, stability and convergence. In Environment II, while the mean values of Crr, Path length and *Nic* remain optimal at $\alpha_{g}$ = 1, the standard deviations of Crr and path length are not the smallest, suggesting that the algorithm maintains the best performance in optimality and convergence, but experiences a slight decline in stability. Overall, the CCPP-TPLP algorithm exhibits the best comprehensive performance when $\alpha_{g}$ is set to 1. Therefore, the value of $\alpha_{g}$ is set to 1 in the subsequent experiments.

As shown in Table 7, applying different perturbation strategies to $POP_{1}$ and $POP_{4}$ affects the algorithm’s performance. In Environment I, perturbation strategy ④ achieves the optimal mean values of Crr, path length and *Nic*, but the standard deviations of Crr and path length are not the smallest. This indicates that the CCPP-TPLP algorithm maintains the best performance in optimality and convergence, though its stability is slightly compromised. In Environment II, perturbation strategy ④ yields optimal mean values and standard deviations of Crr and path length, demonstrating that the algorithm achieves the best performance in both optimality and stability. However, *Nic* result is suboptimal, suggesting a slight impact on convergence speed. Therefore, the perturbation strategy ④ is adopted in the subsequent experiments.

The *t*-test is carried on the ablation study results regarding the greedy factor λ, the degree of greediness $\alpha_{g}$ and perturbation strategy. Specifically, comparative analyses are performed between results of each parameter group (excluding the optimal results) and the optimal results to examine the significance difference under various parameter configurations. The corresponding test outcomes are presented in Table 8, Table 9 and Table 10.

According to Table 8, in Environment I, the *p*-values of *Nic* are all greater than 0.05, indicating that λ has no significant influence on the convergence efficiency of the algorithm However, the *p*-values of path length and Crr are mostly below 0.05 across different values of λ, suggesting that λ significantly influences path length and Crr. In Environment II, the *p*-values for path length and Crr exceed 0.05, implying a weaker impact of λ on these metrics. Conversely, the *p*-value of *Nic* drops below 0.05, demonstrating that λ significantly affects *Nic* in this scenario. Overall, the significant influence law of λ on path length, Crr and *Nic* across environments is not obvious.

It can be seen from Table 9, in Environment I and Environment II, the *p*-values of path length and Crr are all below 0.05, indicating that the value of $\alpha_{g}$ has a significant impact on path length and Crr, while no significant effect on convergence speed is observed.

It can be known from Table 10, compared with perturbation strategy ① and ③, perturbation strategy ④ demonstrates a significant influence on algorithm performance. However, when compared to perturbation strategy ②, the differences caused by perturbation strategy ④ are not statistically significant.

#### 5.2.4. Impact of Population Hierarchical Division Ratio on Algorithm Performance

In order to verify the impact of the population hierarchical division ratio on CCPP-TPLP’s performance, set up a 20 m × 20 m two-dimensional map with 42 grids serving as obstacles for the experiment, and the population size is 50 and the number of iterations is 600. The population is divided into four subgroups, among which the first subgroup $POP_{1}$ is the optimal individual and is a single individual. Thus, the influence of the division ratios of the other three subgroups on the performance of the algorithm is mainly examined. The population is divided by adopting different division ratios respectively, and the experiment is independently repeated 10 times for each division ratio. The results of the optimal path found by the CCPP-TPLP algorithm under different division ratios are recorded and the 10 path results obtained under each division ratio are averaged to obtain the results in Table 11.

It can be observed from Table 11 that the population hierarchical division ratio has a minor influence on the path length. When the division ratio is 2:2:1, Crr is the lowest and the path length is also the lowest. In the subsequent experiments, the population hierarchical division ratio of the three subgroups is set as 2:2:1.

### 5.3. Comparative Experimental Results and Analysis of CCPP-TPLP

**Figure 12:**
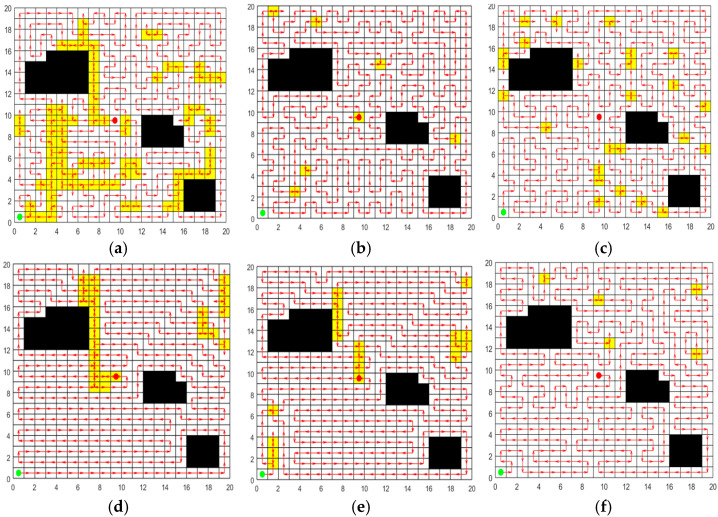
Optimal path obtained by each algorithm in environment I. (**a**) Optimal path of ACO. (**b**) Optimal path of GWO. (**c**) Optimal path of SPBO (**d**) Optimal path of DJAYA. (**e**) Optimal path of DTSA. (**f**) Optimal path of CCPP-TPLP.

**Figure 13:**
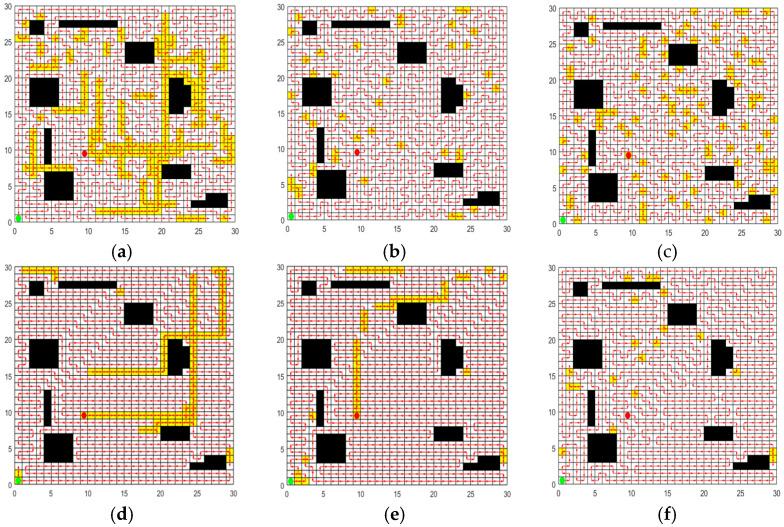
Optimal path obtained by each algorithm in environment II. (**a**) Optimal path of ACO. (**b**) Optimal path of GWO. (**c**) Optimal path of SPBO. (**d**) Optimal path of DJAYA. (**e**) Optimal path of DTSA. (**f**) Optimal path of CCPP-TPLP.

**Figure 14:**
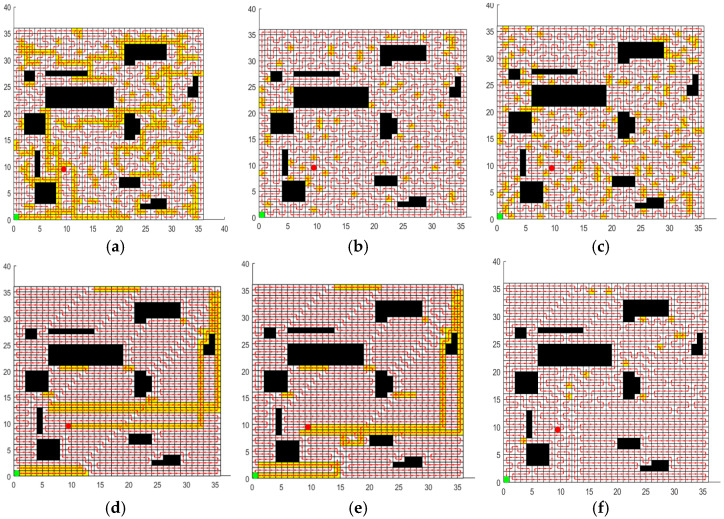
Optimal path obtained by each algorithm in environment III. (**a**) Optimal path of ACO. (**b**) Optimal path of GWO. (**c**) Optimal path of SPBO. (**d**) Optimal path of DJAYA. (**e**) Optimal path of DTSA. (**f**) Optimal path of CCPP-TPLP.

**Figure 15:**
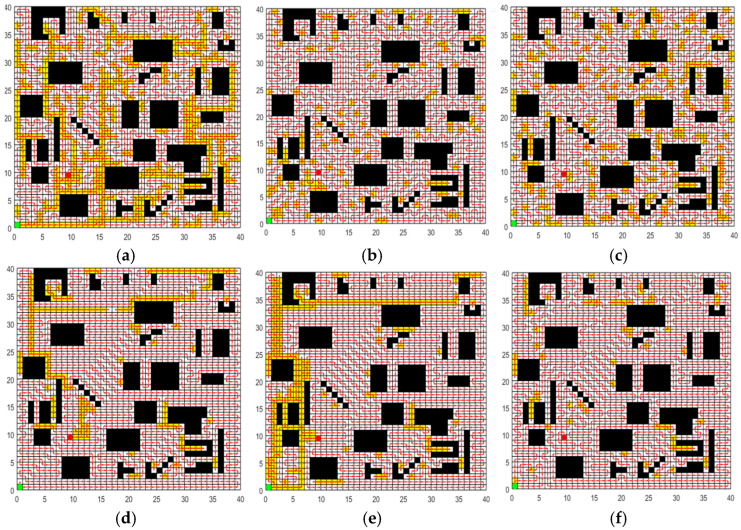
Optimal path obtained by each algorithm in environment IV. (**a**) Optimal path of ACO. (**b**) Optimal path of GWO. (**c**) Optimal path of SPBO. (**d**) Optimal path of DJAYA. (**e**) Optimal path of DTSA. (**f**) Optimal path of CCPP-TPLP.

**Figure 16:**
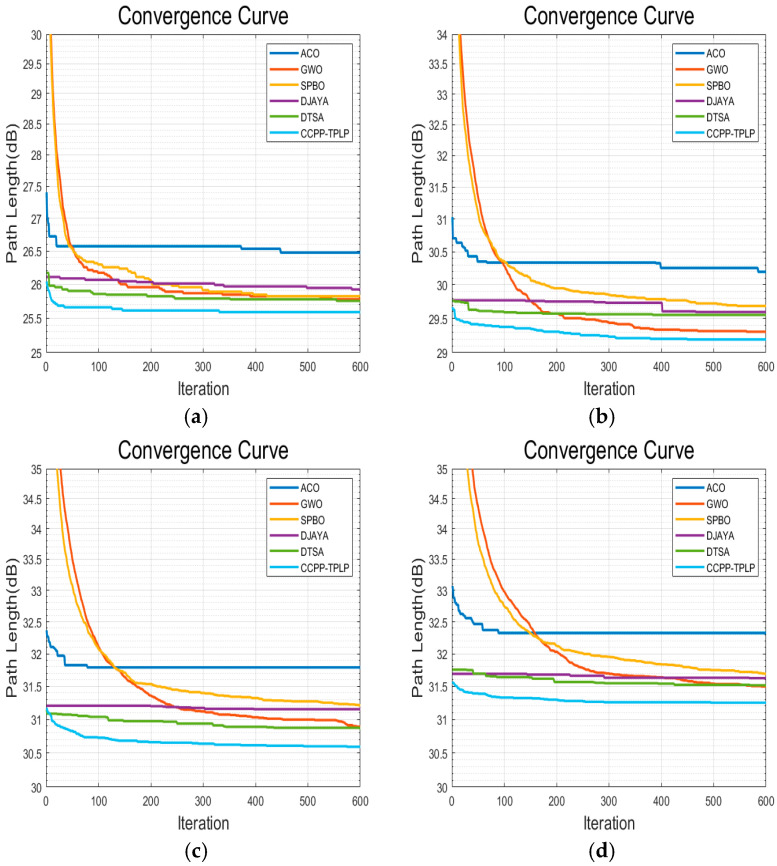
Convergence curves of each algorithm in four map environments. (**a**) Environment I. (**b**) Environment II. (**c**) Environment III. (**d**) Environment IV.

In order to validate the performance of the CCPP-TPLP algorithm proposed in this paper in path planning, the CCPP-TPLP algorithm along with ACO, GWO, SPBO, DJAYA, and DTSA algorithms are respectively tested in four grid maps. The population size is 50, and the number of iterations is 600. Each experiment of different algorithms is independently repeated 10 times. Figure 12, Figure 13, Figure 14 and Figure 15 respectively present the optimal path obtained by each algorithm in environment I, environment II, environment III, and environment IV. The convergence curves of each algorithm in the four map environments are shown in Figure 16.

From Figure 12, Figure 13, Figure 14 and Figure 15 and Table 12, the following conclusions can be drawn:

(1) The CCPP-TPLP algorithm demonstrates significant advantages in the two indicators of Crr and path length. Specifically, in comparison with other algorithms, Crr of the CCPP-TPLP algorithm is reduced by 1.14~16.26%, and the path length is reduced by 1.18~15.75% in environment I. In environment II, Crr of the CCPP-TPLP algorithm is decreased by 3.25~22.99%, and the path length is reduced by 3.16~20.29%. In environment III, Crr of the CCPP-TPLP algorithm is reduced by 4.05~24.68%, and the path length is decreased by 4.02~22.70%. In environment IV, Crr of the CCPP-TPLP algorithm is decreased by 4.35~23.07%, and the path length is reduced by 4.54~22.23%. As the complexity of the environment increases, the performance advantages of the CCPP-TPLP algorithm become more evident.
(2) As shown in Figure 16, both the convergence speed and convergence accuracy of the CCPP-TPLP algorithm are superior to those of the other five comparison algorithms.

### 5.4. CCPP Problem of Electric Tractor

Literature [42] takes the three-dimensional path space as the research object, uses the grid method to store the height information of the three-dimensional path, and combines the rectangular coordinate method and the sequence number method to construct the 2.5-dimensional working environment model of agricultural electric tractor. The composite fitness function is constructed by weighting the flat driving path length and the total height difference. For this model, the CCPP-TPLP algorithm and the five algorithms, i.e., ACO, GWO, SPBO, DJAYA, and DTSA, are respectively employed for optimization.

#### 5.4.1. Settings of Height Information in the Map

**Figure 17:**
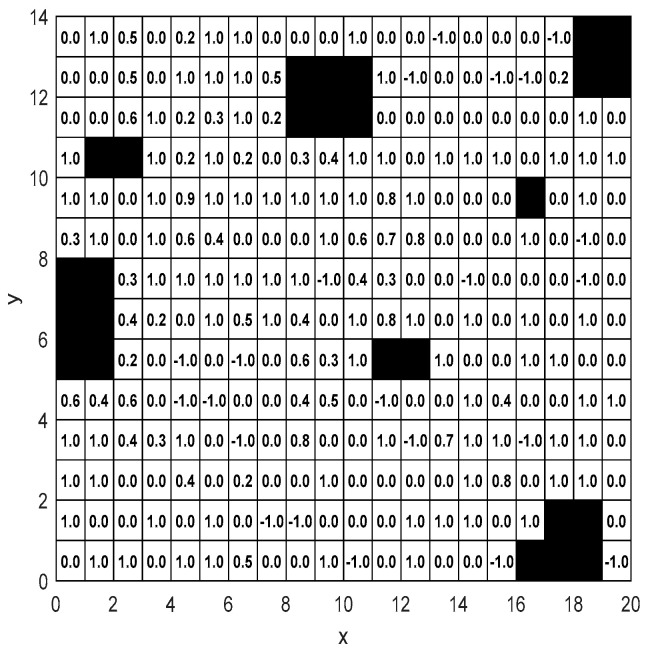
Height information of the map. (The black grids indicate obstacle areas, the white grids represent accessible grids, and the numbers in the grids show their height values.)

Any point on the tractor’s operation path can be expressed as discrete coordinates $\left(x_{i},y_{i},z_{i}\right)$, where $z_{i}$ contains height information. In the rasterized two-dimensional plane model of the tractor working environment, height information $z_{i}$ is set for each grid and stored in a corresponding two-dimensional matrix. The tractor working environment generated in literature [42] is adopted, as shown in Figure 17.

#### 5.4.2. Fitness Function

The fitness function fully considers the energy consumption constraint, which is mainly related to the flat driving path length, the number of turns, and the total total elevation difference. The details are as follows.

The relationship between the length of the flat driving path length b and energy consumption component $e_{1}$ is characterized as Equation (12),

(12) $$e_{1}=F\cos\alpha b$$

Among them, the definitions of each variable are presented in Equation (13), Equation (14) and Equation (15).

(13) $$b=\sum_{i=1}^{K}\sqrt{{\left(x_{i+1}-x_{i}\right)}^{2}+{\left(y_{i+1}-y_{i}\right)}^{2}}$$

(14) F=μmgcosα

(15) $$\alpha =\arctan\frac{h_{i}}{d}(i=1,2,\cdots K)$$

where m represents the mass of the tractor, g indicates the gravitational acceleration, α signifies the slope, μ expresses the friction coefficient, $h_{i}$ denotes the height value of the grid i, and d symbolizes the coordinate distance between two adjacent grids.

The relationship between the total height difference and the energy consumption component $e_{2}$ is defined as Equation (16).

(16) $$e_{2}=mg\sin\alpha \sum_{i=1}^{K}\left|h_{i}-h_{i-1}\right|$$

During the turning process of the tractor, the turning time can be used to indicate the turning consumption of the tractor, and the turning time is directly proportional to the number of turns. Therefore, the tractor’s turning energy consumption can be indicated by the number of turns. The relationship between the number of turns $T_{c}$ and the energy consumption component $e_{3}$ can be expressed by Equation (17).

(17) $$e_{3}=QT_{c}$$

Among them, Q is the energy consumed in one turn, which is calculated by Equations (18) and (19).

(18) $$Q=UI_{t_{1}}$$

(19) $$t_{1}=\frac{\pi d}{2v}$$

where $t_{1}$ represents the time of one turn, U indicates the voltage of the tractor, $I_{t_{1}}$ indicates the current consumed by the tractor in one turn, and v represents the speed of the tractor during turning.

In summary, the total energy consumption of tractor driving can be expressed as Equation (20).

(20) $$E=e_{1}+e_{2}+e_{3}$$

On this basis, the fitness function of the optimization model can be gained by Equation (21).

(21) $$f(X)=\frac{1}{e_{s}e_{1}+e_{h}e_{2}+e_{n}e_{3}}$$

Among them, $e_{s}+e_{h}+e_{n}=1$, it can be observed that the greater the value of the fitness function is, the lower the total energy consumption of the tractor will be.

#### 5.4.3. Experimental Results and Analysis

**Figure 18:**
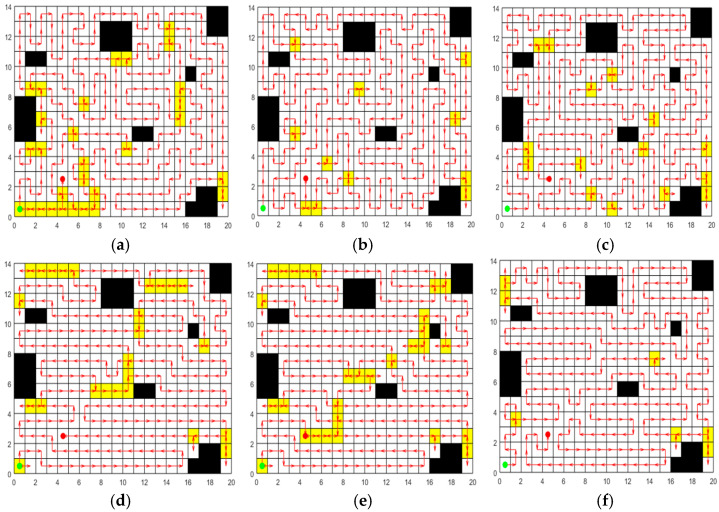
Optimal path obtained by each algorithm in the tractor working map. (**a**) Optimal path of ACO. (**b**) Optimal path of GWO. (**c**) Optimal path of SPBO. (**d**) Optimal path of DJAYA. (**e**) Optimal path of DTSA. (**f**) Optimal path of CCPP-TPLP.

In the experiment, the population size is 50 and the number of iterations is 600, m=0.5 kg, μ=0.25 [43], $g=9.8\;m/s^{2}$, d=1 m, v=0.8 m/s, U=46.2 V, $I_{t_{1}}=50\;A/h$ [44], $e_{s}=0.5$, $e_{h}=0.25$, $e_{n}=0.25$ [45]. Each experiment of different algorithms is independently repeated 20 times. The tractor is capable of traveling only in the four directions: forward, backward, left, and right. The results of 20 repeated independent experiments are statistically presented in Table 13, and the optimal paths obtained by each algorithm are shown in Figure 18.

It can be seen from Table 13 and Figure 18. that the CCPP-TPLP algorithm proposed in this paper has achieved the best results in terms of Crr, path length and fitness value. Particularly in terms of Crr, the CCPP-TPLP algorithm is significantly lower than other algorithms, only 4.74%, which is reduced by 1.05~12.91% compared with other algorithms. The experimental results indicate that the CCPP-TPLP algorithm can acquire the optimal complete coverage path with the lowest energy consumption, the shortest flat driving path length, and the lowest Crr in the CCPP problems of electric tractors.

## 6. Summary and Discussion

In this paper, a complete-coverage path-planning algorithm based on transition probability and learning perturbation operator (CCPP-TPLP) is proposed. Its innovation is demonstrated in two aspects: One is the greedy initialization strategy based on transition probability, and the other is the learning perturbation operation for achieving population update. The shortest distance between each pair of accessible grids in the map is computed, and the grid closer to the current grid is selected as the next path node by employing the greedy strategy, thereby generating higher-quality initial path. On the basis of fitness value ranking, the individuals are classified into different subgroups, and different learning or perturbation operations are adopted for different subgroup to accomplish the update of population. Through ablation experiments, it is confirmed that the greedy initialization strategy based on transition probability enhances the efficiency and quality of initial path planning in terms of path length and Crr. Through the update of learning perturbation operation, a population with a lower Crr and shorter path length can be obtained. Compare with five representative algorithms, CCPP-TPLP demonstrates obvious advantages in the path planning in various map environments. Tests are carried out on the multi-objective weighted CCPP problem of electric tractor, and the findings indicate that CCPP-TPLP could obtain the optimal complete coverage path with the lowest energy consumption, the shortest path length, and the lowest repetition rate. A future research direction lies in exploring the potential of the proposed algorithm in multi-robot cooperative path planning issues, with the aim of achieving parallel computing in a multi-map environment to simultaneously find the optimal path for multiple robots.

## Figures and Tables

**Table 1:** Parameter settings of the comparison algorithms.

Algorithm	Parameter Setting
ACO	The pheromone heuristic factor *α* is 1, the distance heuristic factor *β* is 5, the pheromone heuristic factor is 0.1, and the pheromone enhancement factor is 100
GWO	The convergence factor *a* decreases linearly from 2 to 0
SPBO	The ratio of population distribution is 1:1:1:1
DJAYA	ST1 = 0.5, ST2 = 0.5, the probability of swap transformation swap = 0.2, the probability of shift transformation shift = 0.5, the probability of symmetry transformation symmetry= 0.3
DTSA	ST1 = 0.5, ST2 = 0.5, the probability of swap transformation swap = 0.2, the probability of shift transformation shift = 0.5, the probability of symmetry transformation symmetry= 0.3

**Table 2:** Parameters of four map environments.

Map Environment	Map Size	Proportion of Accessible Area	Quantity of Discrete Obstacles
Environment I	20 m × 20 m	89.50%	3
Environment II	30 m × 30 m	90.00%	7
Environment III	36 m × 36 m	87.50%	11
Environment IV	40 m × 40 m	81.56%	29

**Table 3:** Results of initial paths of the three strategies.

Strategy	Cr	Crr		Path Length	
		Mean	Std.	Mean	Std.
Random initialization strategy	100%	47.54%	0.036	4606.70	54.935
Initialization strategy of ACO	100%	37.57%	0.023	529.50	14.795
Greedy initialization strategy based on transition probability	100%	**9.99%**	**0.008**	**418.60**	**7.950**

Bold indicates the optimal item in this index.

**Table 4:** Statistical results of population updated by the three strategies.

Strategy	Cr	Crr		Path Length	
		Mean	Std.	Mean	Std.
ACO	100%	22.99%	0.017	451.20	4.467
SPBO	100%	22.04%	0.021	441.80	7.194
Learning perturbation operation	100%	**10.60%**	**0.011**	**395.30**	**4.219**

Bold indicates the optimal item in this index.

**Table 5:** The experimental results of different greedy factor λ when $\alpha_{g}$ = 1.

Map Environment	λ	Cr	Crr		Path Length		*Nic*
			Mean	Std.	Mean	Std.	Mean
Environment I	0.1	100%	2.85%	0.008	367.20	3.011	390.10
	0.5	100%	2.51%	0.008	366.40	2.951	364.30
	1	100%	2.57%	0.009	366.20	3.047	284.70
	2	100%	2.57%	0.008	368.20	6.143	396.30
	5	100%	**1.62%**	**0.005**	**362.80**	**1.686**	**290.70**
	10	100%	2.62%	0.008	364.80	2.951	372.40
	20	100%	2.18%	0.014	366.40	5.094	294.50
	50	100%	2.63%	0.006	366.80	2.066	437.60
Environment II	0.1	100%	3.76%	**0.003**	840.40	3.239	514.80
	0.5	100%	3.73%	**0.003**	839.40	**2.836**	511.30
	1	100%	3.92%	0.014	840.80	11.360	472.60
	2	100%	3.80%	0.006	839.80	4.756	497.80
	5	100%	**3.53%**	0.007	**837.60**	5.873	**409.90**
	10	100%	3.65%	0.009	838.60	6.931	473.20
	20	100%	3.95%	0.006	841.00	4.738	484.40
	50	100%	3.58%	0.009	841.20	6.477	533.40

Bold indicates the optimal item in this index.

**Table 6:** The experimental results of different degree of greediness $\alpha_{g}$ when λ = 5.

Map Environment	$\alpha_{g}$	Cr	Crr		Path Length		*Nic*
			Mean	Std.	Mean	Std.	Mean
Environment I	0.5	100%	5.98%	0.011	378.40	3.978	517.30
	0.6	100%	5.25%	0.012	375.80	4.467	491.40
	0.7	100%	5.08%	0.013	375.20	4.541	486.80
	0.8	100%	3.85%	0.013	370.80	4.638	405.90
	0.9	100%	2.85%	0.010	367.20	3.553	388.20
	1	100%	**1.62%**	**0.005**	**362.80**	**1.686**	**290.70**
Environment II	0.5	100%	9.73%	0.009	885.80	7.743	523.60
	0.6	100%	8.88%	**0.006**	881.20	**4.733**	570.00
	0.7	100%	7.48%	0.007	867.00	10.382	514.70
	0.8	100%	6.99%	0.012	865.80	9.818	519.70
	0.9	100%	5.07%	0.013	851.00	11.005	460.00
	1	100%	**3.53%**	0.007	**837.60**	5.873	**409.90**

Bold indicates the optimal item in this index.

**Table 7:** The experimental results of different perturbation strategies.

Map Environment	Perturbation Strategies	Cr	Crr		Path Length		*Nic*
			Mean	Std.	Mean	Std.	Mean
Environment I	$POP_{1}$ and $POP_{4}$: strong perturbation	100%	6.34%	0.014	380.20	4.940	297.40
	$POP_{1}$ and $POP_{4}$: weak perturbation	100%	2.73%	**0.006**	367.20	**2.348**	382.50
	$POP_{1}$: weak perturbation $POP_{4}$: strong perturbation	100%	3.66%	0.010	370.20	3.584	393.10
	$POP_{1}$: strong perturbation $POP_{4}$: weak perturbation	100%	**2.34%**	0.008	**365.40**	2.989	**255.40**
Environment II	$POP_{1}$ and $POP_{4}$: strong perturbation	100%	8.64%	0.016	884.40	13.625	**402.60**
	$POP_{1}$ and $POP_{4}$: weak perturbation	100%	3.69%	0.010	839.00	7.732	466.70
	$POP_{1}$: weak perturbation $POP_{4}$: strong perturbation	100%	5.27%	0.011	851.80	9.259	422.10
	$POP_{1}$: strong perturbation $POP_{4}$: weak perturbation	100%	**3.20%**	**0.008**	**835.20**	**6.811**	407.10

Bold indicates the optimal item in this index.

**Table 8:** The *p*-value of the *t*-test for comparing different λ values with λ = 5.

Map Environment	λ	The *p*-Value of the Path Length	The p-Value of Crr	The *p*-Value of *Nic*
Environment I	0.1	**7.82 × 10^−4^**	**7.83 × 10^−4^**	1.06 × 10^−1^
	0.5	**1.79 × 10^−2^**	**8.44 × 10^−3^**	3.17 × 10^−1^
	1	**6.36 × 10^−3^**	**6.43 × 10^−3^**	9.30 × 10^−1^
	2	**1.52 × 10^−2^**	**3.63 × 10^−3^**	1.30 × 10^−1^
	10	2.53 × 10^−1^	1.81 × 10^−1^	1.10 × 10^−1^
	20	**3.57 × 10^−3^**	**3.62 × 10^−3^**	1.74 × 10^−1^
	50	**1.07 × 10^−4^**	**4.75 × 10^−4^**	1.01 × 10^−1^
Environment II	0.1	2.03 × 10^−1^	3.61 × 10^−1^	**3.55 × 10^−4^**
	0.5	3.94 × 10^−1^	4.47 × 10^−1^	**3.36 × 10^−4^**
	1	4.39 × 10^−1^	4.39 × 10^−1^	**2.44** **× 10^−2^**
	2	3.69 × 10^−1^	3.69 × 10^−1^	**1.32** **× 10^−3^**
	10	7.31 × 10^−1^	7.32 × 10^−1^	**2.08** **× 10^−3^**
	20	1.71 × 10^−1^	1.71 × 10^−1^	**2.57** **× 10^−2^**
	50	2.09 × 10^−1^	8.92 × 10^−1^	**1.75** **× 10^−4^**

Bold indicates the optimal item in this index.

**Table 9:** The *p*-value of the *t*-test for comparing different $\alpha_{g}$ values with $\alpha_{g}$ = 1.

Map Environment	$\alpha_{g}$	The *p*-Value of the Path Length	The p-Value of Crr	The *p*-Value of *Nic*
Environment I	0.5	**1.11 × 10^−9^**	**1.11 × 10^−9^**	**1.53 × 10^−4^**
	0.6	**8.48 × 10^−8^**	**8.80 × 10^−8^**	**1.33 × 10^−3^**
	0.7	**2.07 × 10^−7^**	**2.09 × 10^−7^**	**2.70 × 10^−3^**
	0.8	**7.07 × 10^−5^**	**7.23 × 10^−5^**	**1.61 × 10^−4^**
	0.9	**2.34 × 10^−3^**	**2.37 × 10^−3^**	1.69 × 10^−1^
Environment II	0.5	**6.36 × 10^−11^**	**5.40 × 10^−11^**	5.75 × 10^−2^
	0.6	**1.47 × 10^−11^**	**1.56 × 10^−11^**	**5.28 × 10^−4^**
	0.7	**1.97 × 10^−6^**	**4.68 × 10^−9^**	8.76 × 10^−2^
	0.8	**2.20 × 10^−6^**	**1.85 × 10^−6^**	5.46 × 10^−2^
	0.9	**1.09 × 10^−2^**	**1.06 × 10^−2^**	8.42 × 10^−1^

Bold indicates the optimal item in this index.

**Table 10:** The *p*-value of the *t*-test for comparing different perturbation strategies with perturbation strategy ④.

Map Environment	Perturbation Strategies	The *p*-Value of the Path Length	The p-Value of Crr	The *p*-Value of *Nic*
Environment I	$POP_{1}$ and $POP_{4}$: strong perturbation	**2.03 × 10^−7^**	**4.61 × 10^−7^**	3.65 × 10^−1^
	$POP_{1}$ and $POP_{4}$: weak perturbation	1.51 × 10^−1^	2.48 × 10^−1^	**2.60 × 10^−2^**
	$POP_{1}$: weak perturbation $POP_{4}$: strong perturbation	**4.42 × 10^−3^**	**4.54 × 10^−3^**	**2.25 × 10^−2^**
Environment II	$POP_{1}$ and $POP_{4}$: strong perturbation	**5.31 × 10^−9^**	**1.52 × 10^−8^**	9.53 × 10^−1^
	$POP_{1}$ and $POP_{4}$: weak perturbation	1.93 × 10^−1^	1.82 × 10^−1^	1.65 × 10^−1^
	$POP_{1}$: weak perturbation $POP_{4}$: strong perturbation	**1.66 × 10^−4^**	**1.21 × 10^−4^**	6.59 × 10^−1^

Bold indicates the optimal item in this index.

**Table 11:** The experimental results under different division ratios.

Division Ratio	Cr	Crr		Path Length	
		Mean	Std.	Mean	Std.
0.5:1.5:3	100%	2.63%	0.009	366.20	3.327
1:1:1	100%	2.46%	0.007	365.80	2.394
1:2:1	100%	2.51%	0.007	366.00	2.494
1:2:2	100%	1.84%	**0.006**	363.60	**2.271**
2:1:1	100%	1.62%	0.008	372.80	4.341
2:1:2	100%	1.62%	0.009	362.80	3.327
2:2:1	100%	**1.50%**	0.008	**362.40**	2.836
2.5:1.5:1	100%	2.49%	0.012	366.00	4.320

Bold indicates the optimal item in this index.

**Table 12:** Experimental results of each algorithm in four map environments.

Map Environment	Algorithm	Cr	Crr		Path Length		*Nic*
			Mean	Std.	Mean	Std.	Mean
Environment I	ACO	100%	19.05%	0.032	435.60	12.322	433.40
	GWO	100%	3.93%	0.014	371.40	5.064	494.40
	SPBO	100%	8.10%	0.016	386.00	5.586	567.00
	DJAYA	100%	11.98%	0.025	391.20	12.218	457.40
	DTSA	100%	6.96%	0.021	371.80	4.050	398.90
	CCPP-TPLP	100%	**2.79%**	**0.007**	**367.00**	**2.366**	**312.30**
Environment II	ACO	100%	25.90%	0.028	1044.60	19.323	**280.90**
	GWO	100%	6.16%	0.011	859.80	10.042	543.80
	SPBO	100%	11.85%	0.012	906.00	10.284	572.80
	DJAYA	100%	11.56%	0.018	896.50	18.368	495.60
	DTSA	100%	8.75%	0.021	882.60	17.640	507.80
	CCPP-TPLP	100%	**2.91%**	**0.005**	**832.60**	**4.115**	457.50
Environment III	ACO	100%	27.63%	0.015	1509.00	16.819	**275.70**
	GWO	100%	7.00%	0.013	1215.30	16.680	559.00
	SPBO	100%	15.05%	0.012	1306.40	13.882	568.80
	DJAYA	100%	11.20%	0.010	1271.00	20.044	472.10
	DTSA	100%	11.08%	0.019	1212.20	21.693	500.90
	CCPP-TPLP	100%	**2.95%**	**0.005**	**1166.40**	**5.147**	456.60
Environment IV	ACO	100%	28.74%	0.012	1710.60	9.800	503.50
	GWO	100%	10.02%	0.060	1393.40	16.400	580.20
	SPBO	100%	16.91%	0.010	1479.40	13.500	592.10
	DJAYA	100%	13.56%	0.016	1447.50	21.246	**434.60**
	DTSA	100%	11.82%	0.008	1403.90	19.445	550.20
	CCPP-TPLP	100%	**5.67%**	**0.005**	**1330.20**	**7.083**	510.90

Bold indicates the optimal item in this index.

**Table 13:** Experimental results of each algorithm in the tractor operation environment map.

Algorithm	Cr	Crr		Path Length		Fitness Value	
		Mean	Std.	Mean	Std.	Mean	Std.
ACO	100%	17.65%	0.029	302.50	6.771	3.777 × 10^−3^	7.225 × 10^−5^
GWO	100%	5.79%	0.013	267.70	3.389	3.951 × 10^−3^	7.323 × 10^−5^
SPBO	100%	7.18%	0.015	273.80	3.833	3.876 × 10^−3^	6.854 × 10^−5^
DJAYA	100%	21.44%	0.049	288.30	10.183	4.070 × 10^−3^	**1.986 × 10^−5^**
DTSA	100%	15.22%	0.031	293.90	8.813	4.135 × 10^−3^	8.124 × 10^−5^
CCPP-TPLP	100%	**4.74%**	**0.009**	**265.90**	**2.382**	**4.155 × 10^−3^**	3.091 × 10^−5^

Bold indicates the optimal item in this index.

## Data Availability

Data are contained within the article.
